# Clinical feasibility of duodenum-preserving pancreatic head resection for neuroendocrine tumors of the pancreatic head as an intermediate procedure between enucleation and pancreaticoduodenectomy

**DOI:** 10.20407/fmj.2023-017

**Published:** 2024-05-29

**Authors:** Masahiro Shimura, Hiroyuki Kato, Yukio Asano, Hidetoshi Nagata, Yuka Kondo, Satoshi Arakawa, Daisuke Koike, Takayuki Ochi, Hironobu Yasuoka, Toki Kawai, Takahiko Higashiguchi, Hiroki Tani, Yoshiki Kunimura, Kazuma Horiguchi, Yutaro Kato, Masahiro Ito, Tsunekazu Hanai, Akihiko Horiguchi

**Affiliations:** Department of Gastroenterological Surgery, Fujita Health University, School of Medicine, Nagoya, Aichi, Japan

**Keywords:** Duodenum-preserving pancreatic head resection, Pancreatic neuroendocrine tumors, Surgical alternative

## Abstract

**Objective:**

This study was performed to demonstrate the clinical application of duodenum-preserving pancreatic head resection (DPPHR) as a surgical treatment for pancreatic neuroendocrine tumors (PNETs) in terms of both curability and maintenance of postoperative quality of life.

**Methods:**

Seven patients diagnosed with PNETs underwent DPPHR from January 2011 to December 2021 at our institution. We investigated the clinical relevance of DPPHR based on the patients’ clinicopathological findings.

**Results:**

The median operative time was 492 min, and the median blood loss was 302 g. Postoperative complications were evaluated according to the Clavien–Dindo classification, and postoperative intra-abdominal bleeding was observed in one patient. Pathological examination revealed a World Health Organization classification of G1 in six patients and G2 in one patient. Microvascular invasion was observed in two patients (29%); however, no patients developed lymph node metastasis or recurrence during the follow-up period. A daughter lesion was observed near the primary tumor in one patient. All patients achieved curative resection, and no tumor specimens showed positive margins.

**Conclusions:**

DPPHR facilitates anatomical resection of the pancreatic head in patients with PNETs as well as detailed pathological evaluation of the resected specimen. Therefore, this surgical procedure is an acceptable alternative to pancreaticoduodenectomy or enucleation for patients with PNETs.

## Introduction

Among pancreatic neuroendocrine tumors (PNETs), insulinomas and nonfunctional tumors are low-grade malignancies. Therefore, minimally invasive surgery such as laparoscopic or robotic pancreatic resection is often indicated for these tumors.^1–3^ However, surgeons often encounter a dilemma when performing pancreaticoduodenectomy (PD) for PNETs in the pancreatic head. This is because most PNETs are localized within the pancreas and rarely involve the duodenum or bile duct, making resection of these organs unnecessary. Therefore, several institutions employ enucleation^3,4^ for such cases, which is a tumor-reduction surgery. However, enucleation often results in inadequate resection, making it difficult to evaluate pathologic prognostic factors such as microvascular invasion (MVI).^5^

Duodenum-preserving pancreatic head resection (DPPHR) is a surgical procedure initially reported by Beger et al.^6^ in 1985 for treatment of mass-forming chronic pancreatitis, and the original procedure preserved the parenchyma of the pancreatic rim. In our institution, DPPHR is indicated for patients with low-grade malignant tumors of the pancreatic head; however, only the parenchyma of the pancreatic head is resected, preserving both the duodenum and bile duct.^7^ The greatest advantage of DPPHR is the maintenance of absorption and digestive functions because of preservation of the duodenum. Moreover, DPPHR preserves duodenal hormones and is associated with a low incidence of pancreatic exocrine insufficiency.^8,9^ In addition, compared with subtotal stomach-preserving PD, gastrointestinal reconstruction in DPPHR is simpler and more physiologic, leading to a lower incidence of eating disorders.^10^ Finally, because the common bile duct, duodenum, and papilla of Vater are preserved, there is no reflux of intestinal fluid into the bile duct. This significantly reduces the incidence of postoperative cholangitis.^11,12^

In the present study, we investigated the clinical relevance of DPPHR based on clinicopathological findings in seven patients with PNETs who underwent DPPHR in our department from January 2011 to December 2021. We aimed to demonstrate the clinical application of DPPHR as a surgical treatment for PNETs in terms of both curability and maintenance of postoperative quality of life.

## Methods

### Study participants

Eleven patients were diagnosed with PNETs of the pancreatic head at our institution from January 2011 to December 2021. Among them, seven patients who underwent DPPHR comprised the study population.

### Study design

The surgical technique for DPPHR was described in a previous report.^11^ Briefly, the procedure involves the following steps in order.

∙ Dissection of the gastric and duodenocolic ligaments without Kocher’s maneuver to preserve the duodenal drainage veins
∙ Taping of the common hepatic and gastroduodenal arteries
∙ Division of the pancreatic parenchyma above the portal vein
∙ Preservation of the inferior pancreaticoduodenal artery and anterior superior pancreaticoduodenal artery to the maximum possible extent and ligation of the pancreatic branches of these arteries toward the papilla of Vater
∙ Identification of the bile duct at the upper margin of the pancreas followed by dissection of the pancreatic head from the anterior wall of the bile duct toward the papilla of Vater and confluence of the main pancreatic duct
∙ Ligation of the main pancreatic duct and removal of the pancreatic head
∙ Duct-to-mucosa anastomosis with six to eight interrupted sutures using 5-0 PDS II (Ethicon Inc., Somerville, NJ, USA) for the first-layer anastomosis of the pancreatojejunostomy, followed by the modified Kakita procedure with six sutures using 3-0 Prolene for the second-layer anastomosis of the pancreatojejunostomy^13^
∙ Insertion of a C-tube to avoid delayed biliary leakage

We investigated the clinical relevance of DPPHR based on perioperative outcomes such as the operative time, amount of blood loss, whether preservation of the bile duct was possible, and presence or absence of Clavien–Dindo grade ≥3A postoperative complications. We also evaluated the clinicopathological findings of patients with PNETs, such as the tumor location, tumor diameter, World Health Organization grade, MIB-1 proliferation index, lymph node metastasis, vascular invasion, daughter lesions, tumor recurrence, and patient survival.

## Results

Table 1 summarizes the background characteristics, surgical findings, and postoperative complications of the seven patients who underwent DPPHR for PNETs. The median operative time was 492 min (range, 275–675 min), and the median blood loss was 302 g (range, 15–642 g). We were able to preserve the bile duct in six of seven patients; one patient required bile duct resection. In terms of postoperative complications, one patient developed postoperative intra-abdominal bleeding. All patients achieved curative resection, and none of the resected specimens showed positive margins. None of the patients developed postoperative acute cholangitis, a late postoperative complication frequently seen in patients who undergo PD.^14^

**Figure 1:**
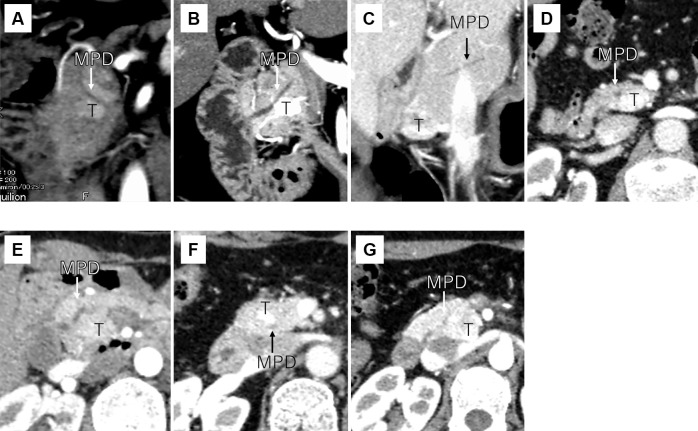
Relationship between tumor location and MPD on enhanced computed tomography (A) Patient 1. (B) Patient 2. (C) Patient 3. (D) Patient 4. (E) Patient 5. (F) Patient 6. (G) Patient 7. T: tumor, MPD: main pancreatic duct

As shown in Table 2, pathological examination revealed a median maximum tumor diameter of 16 mm (range, 8–26 mm), World Health Organization classification^15^ of G1 in six patients and G2 in one, MVI in two patients (29%), and no lymph node metastasis. No patients developed recurrence during the follow-up period. A daughter lesion was observed near the primary tumor in one patient. The primary tumor in all patients was located near the main pancreatic duct as shown in Figure 1.

**Figure 2:**
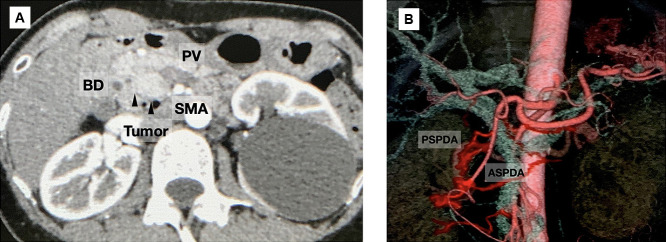
Preoperative images of Patient 5 (A) Preoperative enhanced CT showing a 17-mm well-enhanced tumor (black arrows) in the pancreatic head. (B) CT angiography showing the preoperative arterial anatomy. CT: computed tomography, PV: portal vein, BD: bile duct, SMA: superior mesenteric artery, PSPDA: posterior superior pancreaticoduodenal artery, ASPDA: anterior superior pancreaticoduodenal artery

**Figure 3:**
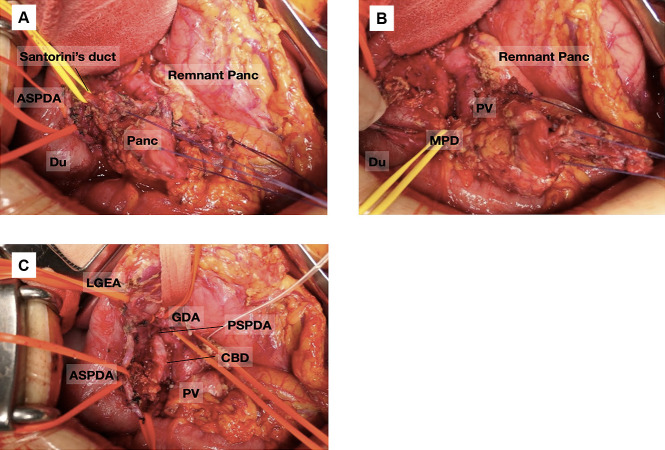
Intraoperative findings of Patient 5 (A) The pancreatic head was mobilized from the retropancreatic uncinate fascia. (B) The pancreatic head was dissected from the duodenum, and the MPD was taped. (C) Images after resection of the pancreatic head. The ASPDA, PSPDA, and CBD were preserved, and the color of the duodenum appears favorable. PV: portal vein, CBD: common bile duct, Du: duodenum, MPD: main pancreatic duct, Panc: pancreas, LGEA: left gastroepiploic artery, GDA: gastroduodenal artery, PSPDA: posterior superior pancreaticoduodenal artery, ASPDA: anterior superior pancreaticoduodenal artery

**Figure 4:**
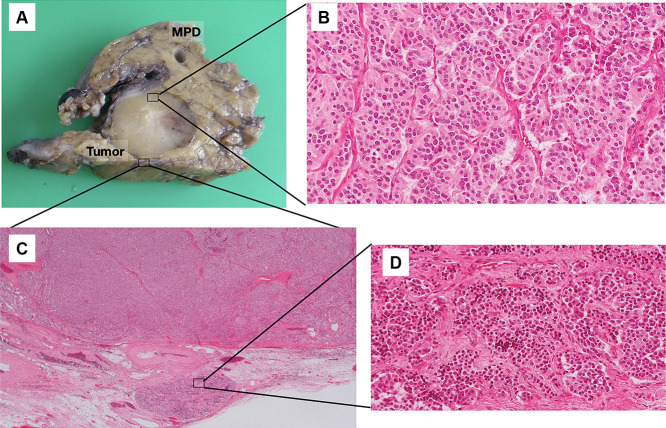
Macroscopic and microscopic findings of the resected specimen in Patient 5. This tumor was diagnosed as a nonfunctional neuroendocrine tumor (A) Macroscopic findings of the tumor, which measured 17×15 mm. (B) Hematoxylin and eosin–stained section of the primary tumor confirming a neuroendocrine tumor. (C) Presence of daughter tumor near the main tumor. (D) Microscopic findings of the daughter tumor.

The clinical course of a representative patient (Patient 5) is herein described. A 51-year-old woman was diagnosed with a PNET by fine-needle biopsy using endoscopic ultrasound at another hospital, and PD was recommended. However, she visited our institution for a second opinion. Preoperative enhanced computed tomography (CT) showed a 17-mm well-enhanced tumor at the pancreatic head with no evidence of bile duct or duodenal invasion (Figure 2A). Peripancreatic lymph node swelling was not observed. CT angiography showed no vascular anomalies (Figure 2B). Thus, we performed DPPHR according to the procedure described above (Figure 3). The resected specimen contained a nonfunctional PNET measuring 17×15 mm. Moreover, a daughter lesion was seen near the primary tumor (Figure 4).

## Discussion

Although this observational study involved only seven patients, we gathered valuable qualitative data, which are summarized as follows. First, 29% (2/7) of the PNETs were found to have MVI and 14% (1/7) had a daughter lesion despite the fact that the tumors appeared localized within the pancreas on preoperative imaging. Second, MVI could be adequately evaluated in these cases, even when anatomical pancreatic head resection was performed with DPPHR. Third, the incidence of short-term complications of DPPHR was acceptable, and no patients developed postoperative acute cholangitis, which commonly occurs after PD.

At our institution, patients with PNETs limited to the pancreas without obvious lymph node metastasis on preoperative imaging are considered good candidates for DPPHR. However, DPPHR is often not indicated if the tumor is adjacent to the bile duct or too large for preservation of the arterial arcade in the pancreatic head. Moreover, DPPHR is not indicated when conventional lymph node dissection is considered necessary based on preoperative CT findings. PD is the only option in such cases.

In the present study, DPPHR was performed in a limited number of patients. However, MVI and a daughter lesion were observed in two and one patient, respectively. Although it is widely accepted that lymph node metastasis is a poor prognostic factor for PNETs,^16,17^ the pathological significance of MVI remains unclear. Nevertheless, Yamaguchi et al.^18^ reported a significantly higher incidence of lymph node metastasis in patients with MVI, and Kim et al.^19^ showed an association between MVI and the prognosis. Unlike enucleation, DPPHR facilitates anatomical resection of the total pancreatic head and can be considered more relevant for surgical removal of PNETs measuring approximately 2 cm in diameter because it allows for proper evaluation of MVI.

Neither of the two patients with MVI in our study developed lymph node metastasis. Compared with PD, the extent of lymph node dissection in DPPHR is restricted; however, lymph nodes around the pancreas, gastroduodenal artery, and common hepatic artery can be dissected. We believe that even if MVI is detected, additional invasive resection similar to PD is not necessary unless peripancreatic lymph node metastasis is observed. In addition, the daughter lesion detected in this study would likely have been undetected if enucleation had been performed, indicating the importance of anatomical resection of the pancreatic head by DPPHR. However, because Kocher’s maneuver is not performed in DPPHR, it is not possible to dissect the lymph nodes in the posterior region of the pancreatic head. Therefore, if lymphadenopathy or metastases in that area are suspected on preoperative imaging, we should cautiously consider the indication for DPPHR.

Preservation of the bile duct by DPPHR is technically challenging and has not been widely performed in recent years. Both short-term complications^20^ and long-term bile duct stenosis^21,22^ are of concern. However, Kato et al.^11^ compared the incidence of short-term complications in patients with low-grade malignant tumors treated with DPPHR and PD and reported no difference in the incidence of short-term complications such as pancreatic fistula. Moreover, they reported a significantly lower incidence of postoperative cholangitis in the long term.^11^ Regarding the long-term nutritional outcomes of DPPHR, Horiguchi et al.^7^ reported that postoperative endocrine and exocrine functions showed significantly greater preservation postoperatively in the DPPHR than PD group. Therefore, DPPHR may contribute to improving the postoperative quality of life of patients with PNETs. However, because this was a retrospective study involving a very small number of patients, further accumulation of cases with long-term follow-up, including evaluation of the prognosis, is needed.

## Conclusions

DPPHR allows anatomical resection of the pancreatic head and detailed pathological evaluation of the resected specimen in patients with PNETs. Moreover, the duodenum and bile duct can be preserved by DPPHR, making this surgical technique less invasive than PD. Therefore, DPPHR might ideally be categorized as an intermediate procedure between enucleation and PD for the treatment of PNETs localized within the pancreas.

## Figures and Tables

**Table1:** Summary of surgical findings and postoperative complications in seven patients who underwent duodenum-preserving pancreatic head resection for pancreatic neuroendocrine tumors

Case no	Age	Sex	Operation time (min)	Blood loss (g)	Preservation of bile duct	Complications CD>III	Postoperative acute cholangitis
1	49	Female	640	309	Possible	No	No
2	36	Female	301	135	Possible	No	No
3	66	Male	413	642	Possible	Abdominal Bleeding	No
4	74	Male	675	302	Impossible	No	No
5	52	Female	492	340	Possible	No	No
6	33	Female	275	15	Possible	No	No
7	52	Female	473	239	Possible	No	No
	52 (33–74)	2:5	492 (275–675)	302 (15–642)	86% (6/7)	14% (1/7)	0% (0/7)

CD: Clavien–Dindo classification

**Table2:** Summary of pathological findings and prognostic outcomes in seven patients who underwent duodenum-preserving pancreatic head resection for pancreatic neuroendocrine tumors

Case No	Tumor Location	Tumor diameter(mm)	WHO grade	MIB1 index (%)	Presence of lymphnode metastasis	Presence of Microvascular invasion	Presence of daughter lesion	Recurrence of tumor	Survivals
1	Ph	17.00	G2	4.2	None	Yes	None	None	47 months alive
2	Ph	11.00	G1	2.1	None	None	None	None	123 months alive
3	Ph	8.00	G1	1.4	None	Yes	None	None	120 months alive
4	Ph	16.00	G1	2.0>	None	None	None	None	25 months alive
5	Ph	17.00	G1	2.7	None	None	Yes	None	25 months alive
6	Ph	26.00	G1	2.0>	None	None	None	None	17 months alive
7	Ph	11.00	G1	1.9	None	None	None	None	12 months alive
		16 (8–26)	G1:6, G2:1	2.1	0%	29%	14%	0%	

WHO: World Health Organization, Ph: pancreatic head

## Availability of Data and Material

All the data generated or analyzed during this study are included in this article.
